# Effects of Liver × receptor agonist treatment on signal transduction pathways in acute lung inflammation

**DOI:** 10.1186/1465-9921-11-19

**Published:** 2010-02-22

**Authors:** Concetta Crisafulli, Emanuela Mazzon, Irene Paterniti, Maria Galuppo, Placido Bramanti, Salvatore Cuzzocrea

**Affiliations:** 1 Department of Clinical and Experimental Medicine and Pharmacology, School of Medicine, University of Messina, Italy; 2 IRCCS Centro Neurolesi "Bonino-Pulejo", Messina, Italy

## Abstract

**Background:**

Liver × receptor α (LXRα) and β (LXRβ) are members of the nuclear receptor super family of ligand-activated transcription factors, a super family which includes the perhaps better known glucocorticoid receptor, estrogen receptor, thyroid receptor, and peroxisome proliferator-activated receptors. There is limited evidence that LXL activation may reduces acute lung inflammation. The aim of this study was to investigate the effects of T0901317, a potent LXR receptor ligand, in a mouse model of carrageenan-induced pleurisy.

**Methods:**

Injection of carrageenan into the pleural cavity of mice elicited an acute inflammatory response characterized by: accumulation of fluid containing a large number of neutrophils (PMNs) in the pleural cavity, infiltration of PMNs in lung tissues and subsequent lipid peroxidation, and increased production of nitrite/nitrate (NOx), tumor necrosis factor-α, (TNF-α) and interleukin-1β (IL-1β). Furthermore, carrageenan induced the expression of iNOS, nitrotyrosine and PARP, as well as induced apoptosis (TUNEL staining and Bax and Bcl-2 expression) in the lung tissues.

**Results:**

Administration of T0901317, 30 min after the challenge with carrageenan, caused a significant reduction in a dose dependent manner of all the parameters of inflammation measured.

**Conclusions:**

Thus, based on these findings we propose that LXR ligand such as T0901317, may be useful in the treatment of various inflammatory diseases.

## Background

Liver × receptor (LXR) is another member of the super family of nuclear hormone receptors, which heterodimerizes with RXR [1]. LXR is activated by natural oxysterols, such as 22(R)-hydroxycholesterol, 24,25(S)-epoxycholesterol, and 27-hydroxycholesterol, and the synthetic compound T0901317 [2], and regulates the intracellular levels of cholesterol through gene induction of enzymes and proteins involved in the cholesterol metabolism and transport [3]. Two LXR subtypes with different tissue distribution have been identified: LXR-α and LXR-β. LXR-α is expressed in macrophages, liver, spleen, kidney, adipose tissue, and small intestine [2], whereas LXR-β is ubiquitously expressed.

In recent years, our understanding of the importance of LXRs has expanded across several fields of patho-physiology. Perhaps best known from a sizeable literature as homeostatic "cholesterol sensors" that drive transcriptional programs promoting cellular cholesterol efflux, "reverse cholesterol transport," and bile acid synthesis [4], more recent roles for LXRs in atherosclerosis [5], renin expression [6], glucose homeostasis [7], innate immunity [8] and in inflammation [9-11] have also been identified.

Various studies have clearly point out that LXRs plays a pivotal role in innate immunity of the macrophage [10]. They inhibit macrophage apoptosis [12] and negatively regulate proinflammatory gene expression (e.g., IL-6, cyclooxygenase 2) induced by LPS and bacteria [13] in macrophages, at least in part through inhibition of NF-κB [14]. LXRs and other nuclear receptors (NRs) such as glucocorticoid receptor (GR) repress overlapping yet distinct sets of proinflammatory genes [15]. Moreover, recent evidence have also clearly demonstrated that endogenous LXR modulation in inflammatory disease states may play a role in pathogenesis [16]. Exploiting these insights, a potential anti-inflammatory therapeutic role for synthetic LXR agonists has recently been described in vivo in a model of dermatitis [13], and data suggest the possibility of therapeutic synergy among NR agonists [15].

To study whether LXR also participates in the acute inflammatory response, mice were injected in the pleural cavity with carrageenan to obtain an acute lung inflammation, usually defined as carrageenan-induced pleurisy. Carrageenan-induced inflammation (paw edema or pleurisy) is a model of local acute inflammation commonly used to evaluate activity of anti-inflammatory drugs [17] and useful to assess the contribution of cells and mediators to the inflammatory process [18]. The initial phase of carrageenan-induced pleurisy (0-1 h) has been attributed to the release of histamine, 5-hydroxytryptamine and bradykinin, followed by a late phase (1-6 h) mainly sustained by PG release due to the induction of cyclooxygenase 2 (COX-2) in the tissues [19]. PMNs moving out of the circulation into the inflamed tissue have a key function in the breakdown and remodeling of injured tissue [20,21]. Moreover, macrophages participate in the progression of experimental pleurisy producing pro-inflammatory cytokines such as TNFα and IL-1β.

In the present study, to explore further the possible role of LXR in the modulation of different inflammatory conditions *in vivo*, the effects of the potent LXR receptor ligand T0901317, were observed on (i) polymorphonuclear (PMN) infiltration (assessing myeloperoxidase [MPO] activity), (ii) lipid peroxidation (malondialdehyde [MDA] levels), (iii) pro-inflammatory cytokines (TNF-α and IL-1β), (iv) nitration of tyrosine residues as an indicator of peroxynitrite (by immunohistichemistry), (v) inducible nitric oxide synthase (iNOS) expression, (vi) NF-κB expression, (vii) apoptosis (FAS-ligand and TUNEL staining), (viii) Bax and Bcl-2 expression, and (ix) lung damage (histology).

## Methods

### Animals

Male CD mice (weight 20-25 g; Harlan Nossan, Milan, Italy) were used in these studies. The animals were housed in a controlled environment and provided with standard rodent chow and water. Animal care was in compliance with Italian regulations on the protection of animals used for experimental and other scientific purposes (D.M. 116192) as well as with EEC regulations (O.J. of E.C. L358/1 12/18/1986).

### Carrageenan-induced pleurisy

Carrageenan-induced pleurisy was induced as previously described [22]. Mice were anaesthetized with isoflurane and subjected to a skin incision at the level of the left sixth intercostals space. The underlying muscle was dissected and saline (0.1 ml) or saline containing 2% λ-carrageenan (0.1 ml) was injected into the pleural cavity. The skin incision was closed with a suture and the animals were allowed to recover. At 4 h after the injection of carrageenan, the animals were killed by inhalation of CO_2_. The chest was carefully opened and the pleural cavity rinsed with 1 ml of saline solution containing heparin (5 U ml^-1^) and indomethacin (10 μg ml^-1^). The exudate and washing solution were removed by aspiration and the total volume measured. Any exudate, which was contaminated with blood, was discarded. The amount of exudate was calculated by subtracting the volume injected (1 ml) from the total volume recovered. The leukocytes in the exudate were suspended in phosphate-buffer saline (PBS) and counted with an optical microscope in a Burker's chamber after Blue Toluidine staining.

### Experimental Design

Mice were randomized into 4 groups. Sham animals were subjected to the surgical procedure alone, receiving a bolus injection of saline (1 ml/kg i.p.) instead of carrageenan, and treated 30 min after with either vehicle (saline 1 ml/kg i.p.) or T0901317 (20, 10 and 5 mg/kg, i.p.). The remaining mice were subjected to carrageenan-induced pleurisy (as described above) and treated with an i.p. bolus of vehicle (saline1 ml/kg) or 20, 10 and 5 mg/kg T0901317. N = 10 per group. The doses of T0901317 (20, 10 and 5 mg/kg, i.p.) used here were based on previous *in vivo *studies [23,24]

### Histological examination

Lung tissues samples were taken 4 h after injection of carrageenan. Lung tissues samples were fixed for 1 week in 10% (w/v) PBS-buffered formaldehyde solution at room temperature, dehydrated using graded ethanol and embedded in Paraplast (Sherwood Medical, Mahwah, NJ, USA). Sections were then deparaffinized with xylene, stained with hematoxylin and eosin. All sections were studied using Axiovision Zeiss (Milan, Italy) microscope.

### Measurement of cytokines

TNF-α and IL-1β levels were evaluated in the exudate 4 h after the induction of pleurisy by carrageenan injection as previously described [25]. The assay was carried out using a colorimetric commercial ELISA kit (Calbiochem-Novabiochem Corporation, Milan, Italy).

### Measurement of nitrite-nitrate concentration

Total nitrite in exudates, an indicator of nitric oxide (NO) synthesis, was measured as previously described [26]. Briefly, the nitrate in the sample was first reduced to nitrite by incubation with nitrate reductase (670 mU/ml) and β-nicotinamide adenine dinucleotide 3'-phosphate (NADPH) (160 μM) at room temperature for 3 h. The total nitrite concentration in the samples was then measured using the Griess reaction, by adding 100 μl of Griess reagent (0.1% w/v) naphthylethylendiamide dihydrochloride in H_2_O and 1% (w/v) sulphanilamide in 5% (v/v) concentrated H_3_PO_4_; vol. 1:1) to the 100 μl sample. The optical density at 550 nm (OD_550_) was measured using ELISA microplate reader (SLT-Lab Instruments, Salzburg, Austria). Nitrite concentrations were calculated by comparison with OD_550 _of standard solutions of sodium nitrite prepared in H_2_O.

### Immunohistochemical localization of iNOS, IL-1β, TNF-α, nitrotyrosine, PAR, Fas ligand, Bax and Bcl-2

At the end of the experiment, the tissues were fixed in 10% (w/v) PBS-buffered formaldehyde and 8 μm sections were prepared from paraffin embedded tissues. After deparaffinization, endogenous peroxidase was quenched with 0.3% (v/v) hydrogen peroxide in 60% (v/v) methanol for 30 min. The sections were permeabilized with 0.1% (w/v) Triton X-100 in PBS for 20 min. Non-specific adsorption was minimized by incubating the section in 2% (v/v) normal goat serum in PBS for 20 min. Endogenous biotin or avidin binding sites were blocked by sequential incubation for 15 min with biotin and avidin, respectively. Sections were incubated overnight with anti-iNOS (1:500, Transduction Laboratories in PBS, v/v), anti-nitrotyrosine rabbit polyclonal antibody (Upstate, 1:500 in PBS, v/v), anti-PAR antibody (BioMol, 1:200 in PBS, v/v), anti-FAS ligand antibody (Santa Cruz Biotechnology, 1:500 in PBS, v/v), anti-TNF-α ligand antibody (Santa Cruz Biotechnology, 1:500 in PBS, v/v), anti-IL-1β ligand antibody (Santa Cruz Biotechnology, 1:500 in PBS, v/v), anti-Bax antibody (Santa Cruz Biotechnology, 1:500 in PBS, v/v) or with anti-Bcl-2 polyclonal antibody (Santa Cruz Biotechnology, 1:500 in PBS, v/v). Sections were washed with PBS, and incubated with secondary antibody. Specific labeling was detected with a biotin-conjugated goat anti-rabbit IgG and avidin-biotin peroxidase complex (Vector Laboratories, DBA).

In order to confirm that the immunoreaction for the nitrotyrosine was specific some sections were also incubated with the primary antibody (anti-nitrotyrosine) in the presence of excess nitrotyrosine (10 mM) to verify the binding specificity. To verify the binding specificity for iNOS, IL-1β, TNF-α, PAR, Fas ligand, Bax and Bcl-2, some sections were also incubated with only the primary antibody (no secondary) or with only the secondary antibody (no primary). In these situations no positive staining was found in the sections indicating that the immunoreaction was positive in all the experiments carried out.

### Myeloperoxidase (MPO) activity

MPO activity, an indicator of PMN accumulation, was determined as previously described [27]. At the specified time following injection of carrageenan, lung tissues were obtained and weighed, each piece homogenized in a solution containing 0.5% (w/v) hexadecyltrimethyl-ammonium bromide dissolved in 10 mM potassium phosphate buffer (pH 7) and centrifuged for 30 min at 20,000 × g at 4°C. An aliquot of the supernatant was then allowed to react with a solution of tetramethylbenzidine (1.6 mM) and 0.1 mM hydrogen peroxide. The rate of change in absorbance was measured spectrophotometrically at 650 nm. MPO activity was defined as the quantity of enzyme degrading 1 μmol of peroxide min^-1 ^at 37°C and was expressed in milliunits per gram weight of wet tissue.

### Malondialdehyde (MDA) measurement

MDA levels in the lung tissue were determined as an indicator of lipid peroxidation as previously described [28]. Lung tissue collected at the specified time, was homogenized in 1.15% (w/v) KCl solution. A 100 μl aliquot of the homogenate was added to a reaction mixture containing 200 μl of 8.1% (w/v) SDS, 1.5 ml of 20% (v/v) acetic acid (pH 3.5), 1.5 ml of 0.8% (w/v) thiobarbituric acid and 700 μl distilled water. Samples were then boiled for 1 h at 95°C and centrifuged at 3,000 × g for 10 min. The absorbance of the supernatant was measured using spectrophotometry at 650 nm.

### Western blot analysis for IκB-α, NF-κB p65, Bax, Bcl-2, and iNOS

Cytosolic and nuclear extracts were prepared with slight modifications. Briefly, lung tissues from each mouse were suspended in extraction Buffer A containing Hepes 10 mM, KCl 10 mM, EDTA 0.1 mM, EGTA 0.1 mM, DTT 1 mM, PMSF 0.5 mM, pepstatin A 3 μg/ml, leupeptin 2 μg/ml, Trypsin inhibitor 15 μg/ml, Benzamidina 40 μM, homogenized at the highest setting for 2 min, and centrifuged at 13,000 × g for 3 min at 4°C. Supernatants represented the cytosolic fraction. The pellets, containing enriched nuclei, were re-suspended in Buffer B containing Hepes 20 mM, MgCl_2 _1.5 mM, NaCl 0.4 M, EGTA 1 mM, EDTA 1 mM, DTT 1 mM, PMSF 0,5 mM, pepstatin A 3 μg/ml, leupeptin 2 μg/ml, Trypsin inhibitor 15 μg/ml, Benzamidina 40 μM, NONIDET P40 1%, Glycerol 20%. After centrifugation 10 min at 13,000 × g at 4°C, the supernatants containing the nuclear protein were stored at -80 for further analysis. The levels of IκB-α, iNOS, Bax and Bcl-2 were quantified in cytosolic fraction from lung tissue collected 4 h after carrageenan administration, while NF-κB p65 levels were quantified in nuclear fraction. Protein concentration in cell lysates was determined by Bio-Rad Protein Assay (BioRad, Richmond CA) and 50 μg of cytosol and nuclear extract from each sample was analyzed. Proteins were separated by a 12% SDS-polyacrylamide gel electrophoresis and transferred on PVDF membrane (Hybond-P, Amershan Biosciences, UK). The membrane was blocked with 0.1% TBS-Tween containing 5% non fat milk for 1 h at room temperature and subsequently probed with specific Abs IκB-α (Santa Cruz Biotechnology, 1:1000), or anti-Bax (1:500; Santa Cruz Biotechnology), or anti-Bcl-2 (1:500; Santa Cruz Biotechnology), or anti-iNOS (1:1000; Transduction) or anti-NF-kB p65 (1:1000; Santa Cruz Biotechnology) in 1× PBS, 5% w/v non fat dried milk, 0.1% Tween-20 (PMT) at 4°C, overnight. Membranes were incubated with peroxidase-conjugated bovine anti-mouse IgG secondary antibody or peroxidase-conjugated goat anti-rabbit IgG (1:2000, Jackson ImmunoResearch, West Grove, PA) for 1 h at room temperature. To ascertain that blots were loaded with equal amounts of proteic lysates, they were also incubated in the presence of the antibody against β-actin protein (1:10,000 Sigma-Aldrich Corp.) and anti-Lamin B1 (1:10,000 Sigma-Aldrich Corp.). Protein bands were detected with SuperSignal West Pico Chemioluminescent (PIERCE). The relative expression of the protein bands of IκB-α (~37 kDa), NF-kB p65 (~65 kDa), Bax (~23 kDa), Bcl-2 (~29 kDa) iNOS (~130 kDa), was quantified by densitometric scanning of the X-ray films with GS-700 Imaging Densitometer (GS-700, Bio-Rad Laboratories, Milan, Italy) and a computer program (Molecular Analyst, IBM), and standardized for densitometric analysis to β-actin and Lamin B1 protein levels.

### Terminal Deoxynucleotidyltransferase-Mediated UTP End Labeling (TUNEL) Assay

TUNEL assay was conducted by using a TUNEL detection kit according to the manufacturer's instructions (Apotag, HRP kit DBA, Milan, Italy). Briefly, sections were incubated with 15 μg/ml proteinase K for 15 min at room temperature and then washed with PBS. Endogenous peroxidase was inactivated by 3% H_2_O_2 _for 5 min at room temperature and then washed with PBS. Sections were immersed in terminal deoxynucleotidyltransferase (TdT) buffer containing deoxynucleotidyl transferase and biotinylated dUTP in TdT buffer, incubated in a humid atmosphere at 37°C for 90 min, and then washed with PBS. The sections were incubated at room temperature for 30 min with anti-horseradish peroxidase-conjugated antibody, and the signals were visualized with diaminobenzidine.

### Materials

Unless otherwise stated, all compounds were obtained from Sigma-Aldrich Company Ltd. (Poole, Dorset, U.K.). T0901317 was obtained from Cayman Chemical (Michigan, USA). All other chemicals were of the highest commercial grade available. All stock solutions were prepared in non-pyrogenic saline (0.9% NaCl; Baxter, Italy, UK).

### Statistical evaluation

All values in the figures and text are expressed as mean ± standard error (s.e.m.) of the mean of *n *observations. For the *in vivo *studies *n *represents the number of animals studied. In the experiments involving histology or immunohistochemistry, the figures shown are representative of at least three experiments (histological or immunohistochemistry coloration) performed on different experimental days on the tissue sections collected from all the animals in each group. The results were analyzed by one-way ANOVA followed by a Bonferroni *post-hoc *test for multiple comparisons. A *p*-value less than 0.05 were considered significant and individual group means were then compared with Student's unpaired t test. A *P*-value of less than 0.05 was considered significant.

## Results

### Effects of T0901317 on carrageenan-induced pleurisy

**Figure 1:**
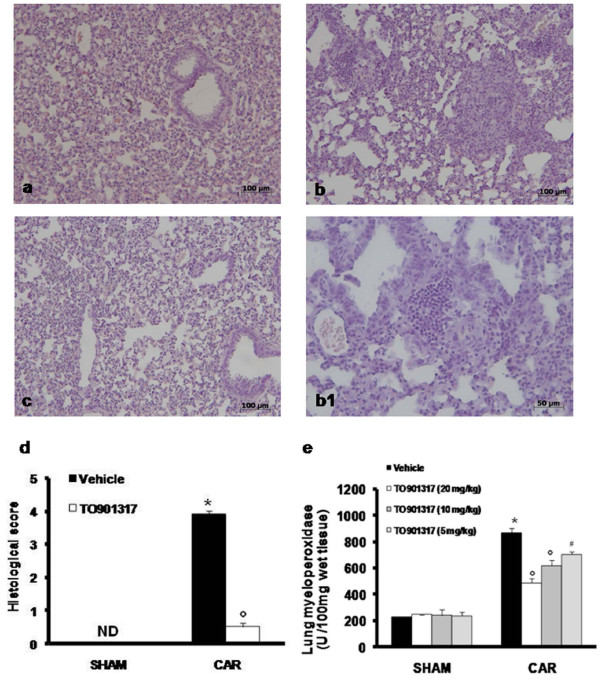
**Effect of T0901317 on histological alterations of lung tissue 4 h after carrageenan-induced injury and on PMN infiltration in the lung**. Lung sections taken from carrageenan-treated mice pre-treated with vehicle demonstrated edema, tissue injury (b, d) as well as infiltration of the tissue with neutrophils (see b1). Carrageenan-treated animals pre-treated with T0901317 (20 mg/kg i.p.) (c, d) demonstrated reduced lung injury and neutrophil infiltration. Original magnification: × 125. Section from a sham animals demonstrating the normal architecture of the lung tissue (a, d). The figure is representative of at least 3 experiments performed on different experimental days. MPO activity, index of PMN infiltration, was significantly elevated at 4 h after carrageenan (CAR) administration in vehicle-treated mice (e), if compared with sham mice (e). T0901317 significantly reduced in a dose dependent manner MPO activity in the lung (e). The figure is representative of at least 3 experiments performed on different experimental days. Data are expressed as mean ± s.e.m. from n = 10 mice for each group. ND: not detectable. *P < 0.01 versus sham group. °*P *< 0.01 versus carrageenan.

When compared to lung sections taken from saline-treated animals (sham group Fig. 1a, d), histological examination of lung sections taken from mice treated with carrageenan revealed significant tissue damage and edema (Fig. 1b, see densitometry analysis 1d), as well as infiltration of neutrophils (PMNs) within the tissues (see Fig 1b1, see densitometry analysis 1d). T0901317 (20 mg/kg) reduced the degree of lung injury (Fig. 1c, d). Furthermore, injection of carrageenan elicited an acute inflammatory response characterized by the accumulation of fluid (edema) in the pleural cavity (Table 1) containing large amounts of PMNs (Table 1). Treatment with T0901317 attenuated in a dose dependent manner carrageenan-induced edema formation and PMN infiltration (Table 1).

**Table 1:** Effect of T0901317 on Carrageenan(CAR)-induced inflammation, TNF-α, IL-1β and Nitrite Nitrate production in the pleural exudate

	Volume Exudate (ml)	PMNs infiltration (million cells/mouse)	TNF-α (pg/ml)	IL-1β (pg/ml)	Nitrite/nitrate (nmol/mouse)
**Sham + Vehicle**	0.06 ± 0.03	0.4 ± 0.12	8.0 ± 0.6	6.0 ± 1.3	12 ± 1
					
**Sham + T0901317** **(20 mg/kg)**	0.07 ± 0.05	0.5 ± 0.18	9.0 ± 0.7	7.0 ± 2.2	11 ± 1.2
					
**CAR + Vehicle**	1.2 ± 0.12*	9.5 ± 0.9*	55 ± 4.5*	151 ± 12*	135 ± 18*
					
**CAR + T0901317** **(20 mg/kg)**	0.20 ± 0.1°	2.5 ± 0.18°	19 ± 1.5°	35 ± 7.5°	40 ± 3.5°
					
**CAR + T0901317** **(10 mg/kg)**	0.41 ± 0.14°	4.5 ± 0.22°	29 ± 1.6°	65 ± 6.8°	74 ± 4.5°
					
**CAR + T0901317** **(5 mg/kg)**	0.90 ± 0.15^#^	7.5 ± 0.35^#^	32 ± 2.5^#^	95 ± 4.5^#^	100 ± 2.4^#^

Data are means ± s.e. means of 10 mice for each group. *P < 0.01 versus sham. °P < 0.01 versus carrageenan ^#^P < 0.05 versus carrageenan.

The pleural infiltration with PMN appeared to correlate with an influx of leukocytes into the lung tissue, thus we investigated the effect of T0901317 on neutrophil infiltration by measurement of myeloperoxidase activity. Myeloperoxidase activity was significantly elevated at 4 h after carrageenan administration in vehicle-treated mice (Fig. 1e). Treatment with T0901317 significantly attenuated in a dose dependent manner neutrophil infiltration into the lung tissue (Fig. 1e).

### Effects of T0901317 on carrageenan-induced NO production

**Figure 2:**
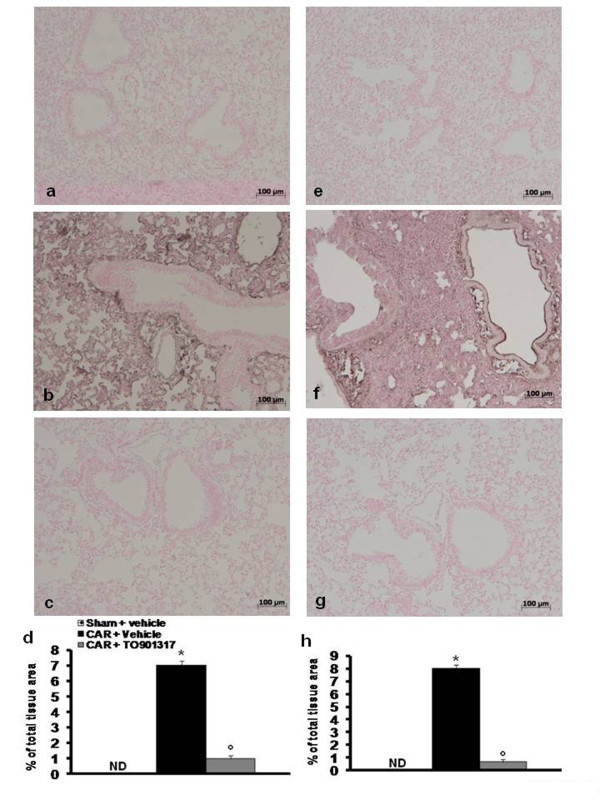
**Effect of T0901317 on carrageenan-induced iNOS expression and NO formation in the lung**. Lung sections taken from carrageenan-treated mice pre-treated with vehicle showed positive staining for iNOS, localized mainly in inflammatory cells (b, e). The degree of positive staining for iNOS was markedly reduced in tissue sections obtained from mice pre-treated with 20 mg/kg T0901317 (c, e). Original magnification: × 125. Lung sections taken from sham mice showed no staining for iNOS (a, e). The figure is representative of at least 3 experiments performed on different experimental days. A significant increase in iNOS (d, d1) expression, assayed by Western blot analysis, was detected in lungs obtained from mice subjected to carrageenan-induced pleurisy, if compared with lung from sham mice (d, d1). Pre-treatment with T0901317 20 mg/kg significantly attenuated iNOS (d, d1) expression in the lung tissues. A representative blot of lysates obtained from 5 animals per group is shown and densitometry analysis of all animals is reported. The results in panel d1 are expressed as mean ± s.e.m. from n = 5/6 lung tissues for each group. ND: not detectable. *P < 0.01 versus sham group. °*P *< 0.01 versus carrageenan.

No positive staining for iNOS was observed in the lung tissues obtained from the sham group (Fig. 2a, see densitometry analysis 2e). Immunohistochemical analysis of lung sections obtained from carrageenan-treated mice revealed positive staining for iNOS (Fig. 2b, see densitometry analysis 2e). T0901317 (20 mg/kg) treatment significantly attenuated this iNOS expression (Fig. 2c, see densitometry analysis 2e). A significant increase in iNOS expression 4 h after carrageenan injection, as assayed by Western blot analysis, was also detected in lungs obtained from mice subjected to carrageenan-induced pleurisy (Fig. 2d see densitometry analysis 2d1). T0901317 (20 mg/kg) treatment significantly attenuated this iNOS expression (Fig. 2d see densitometry analysis 2d1). NO levels were also significantly increased in the exudate obtained from mice administered carrageenan (Table 1). Treatment of mice with T0901317 significantly reduced in a dose dependent manner NO exudates levels (Table 1). No significant reduction of NO exudates levels was found in the sham animal.

### Effects of T0901317 on carrageenan-induced nitrotyrosine formation, lipid peroxidation and PARP activation

**Figure 3:**
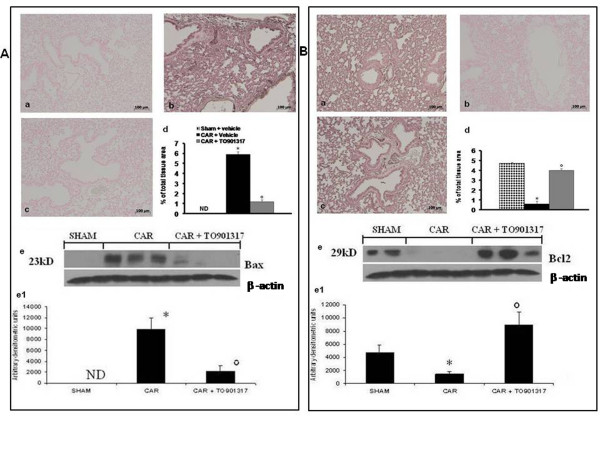
**Effect of T0901317 on carrageenan-induced nitrotyrosine formation and lipid peroxidation and PARP activation in the lung**. No staining for nitrotyrosine is present in lung section from sham mice (a, g). Lung sections taken from carrageenan-treated mice pre-treated with vehicle showed positive staining for nitrotyrosine, localized mainly in inflammatory cells (b, g). There was a marked reduction in the immunostaining for nitrotyrosine in the lungs of carrageenan-treated mice pre-treated with 20 mg/kg T0901317 (c, g). Malondialdehyde (MDA) levels, an index of lipid peroxidation, were significantly increased in lung tissues 4 h after carrageenan (CAR) administration (h), if compared with lung from sham mice (h). T0901317 significantly reduced in a dose dependent manner the carrageenan-induced elevation of MDA tissues levels (h). Lung sections taken from carrageenan-treated mice pre-treated with vehicle showed positive staining for PAR (e, g). There was a marked reduction in the immunostaining for PAR in the lungs of carrageenan-treated mice pre-treated with 20 mg/kg T0901317 (f, g). Lung section from sham mice showed no staining for PAR (d, g). The figure is representative of at least 3 experiments performed on different experimental days. Data are expressed as mean ± s.e.m. from n = 10 mice for each group. ND: not detectable *P < 0.01 versus sham group. °*P *< 0.01 versus carrageenan.

Immunohistochemical analysis of lung sections obtained from mice treated with carrageenan revealed positive staining for nitrotyrosine (Fig. 3b, see densitometry analysis 3g). In contrast, no positive staining for nitrotyrosine was found in the lungs of carrageenan-treated mice, which had been treated with T0901317 (20 mg/kg) (Fig. 3c, see densitometry analysis 3g). In addition, at 4 hours after carrageenan-induced pleurisy, MDA levels were also measured in the lungs as an indicator of lipid peroxidation. As shown in Figure 3h, MDA levels were significantly increased in the lungs of carrageenan-treated mice. Lipid peroxidation was significantly attenuated in a dose dependent manner by the intraperitoneal injection of T0901317 (Fig. 3h). At the same time point (4 h after carrageenan administration), lung tissue sections were taken in order to determine the immunohistological staining for poly ADP-ribosylated proteins (an indicator of PARP activation). A positive staining for the PAR (Fig. 3e, see densitometry analysis 3g) was found primarily localized in the inflammatory cells present in the lung tissue from carrageenan-treated mice. T0901317 treatment reduced the degree of PARP activation (Figure 3f, see densitometry analysis 3g). Please note that there was no staining for either nitrotyrosine (Fig. 3a, see densitometry analysis 3g) or PAR (Fig. 3d, see densitometry analysis 3g) in lung tissues obtained from the sham group of mice.

### Effects of T0901317 on the release of pro-inflammatory cytokine induced by carrageenan

**Figure 4:**
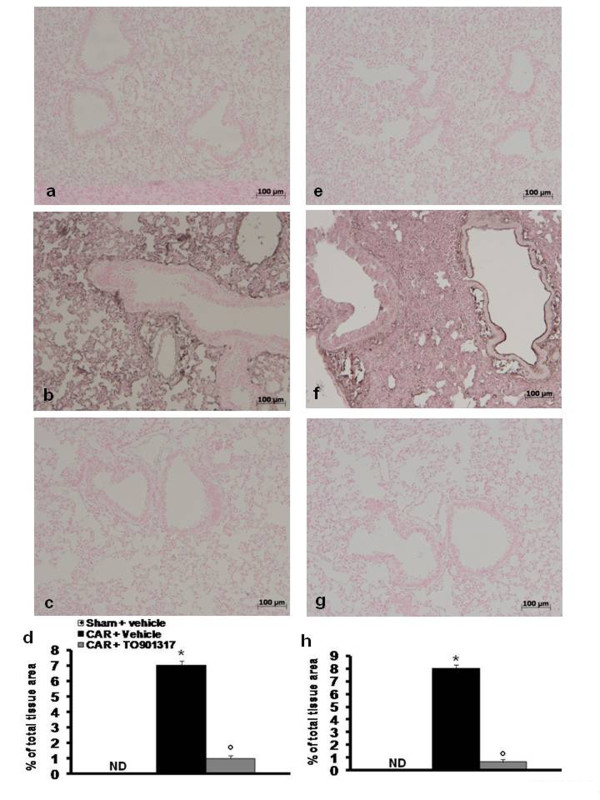
**Effect of T0901317 on carrageenan-induced pro-inflammatory cytokine release in the lung**. Lung sections taken from carrageenan-treated mice pre-treated with vehicle showed positive staining for TNF-α and IL-1β (b, d and f, h). There was a marked reduction in the immunostaining for TNF-α and IL-1β in the lungs of carrageenan-treated mice pre-treated with 20 mg/kg T0901317 (c, d and g, h). No staining for either TNF-α (a, d) or IL-1β (e, h) in lung tissues obtained from the sham group of mice. The figure is representative of at least 3 experiments performed on different experimental days. ND: not detectable. Data are expressed as mean ± s.e.m. from n = 10 mice for each group. *P < 0.01 versus sham group. °*P *< 0.01 versus carrageenan.

When compared to sham animals, injection of carrageenan resulted in an increase in the levels of TNF-α and IL-1β in the pleural exudates (Table 1). The release of TNF-α and IL-1β was significantly attenuated in a dose dependent manner by treatment with T0901317 (Table 1). Therefore, we also evaluate the TNF-α and IL-1β expression in the lung tissues by immunohistochemical detection. Tissue sections obtained from vehicle-treated animals at 4 h after carrageenan injection demonstrate positive staining for TNF-α mainly localized in the infiltrated inflammatory cells, pneumocytes as well as in vascular wall (Fig. 4b, see densitometry analysis 4d). In contrast, no staining for TNF-α was found in the lungs of carrageenan-treated mice that had been treated with T0901317 (Fig. 4c, see densitometry analysis 4d). Similarly, at 4 hours after carrageenan injection, positive staining for IL-1β mainly localized in the infiltrated inflammatory cells was observed in lung tissue sections obtained from vehicle-treated animals (Fig. 4f, see densitometry analysis 4h). T0901317 treatment reduced the degree of IL-1β expression (Fig. 4g, see densitometry analysis 4h). Please note that there was no staining for either TNF-α (Fig. 4a, see densitometry analysis 4d) or IL-1β (Fig. 4e, see densitometry analysis 4h) in lung tissues obtained from the sham group of mice.

### Effect of T0901317 on IκB-α degradation and NF-κB p65 activation

**Figure 5:**
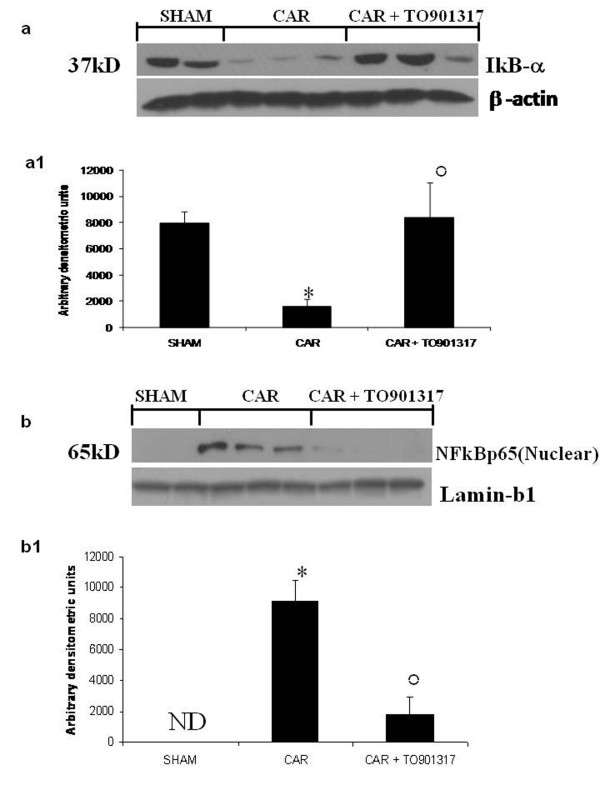
**Representative Western blots showing the effects of T0901317 on IκB-α degradation and nuclear NF-κ Bp65 expression after carrageenan (CAR) injection**. Basal expression of IκB-α was detected in lung samples from sham-treated animals, whereas IκB-α levels were substantially reduced in lung tissues obtained from vehicle-treated animals at 4 h after carrageenan injection (a, a1). T0901317 (20 mg/kg) treatment prevented carrageenan-induced IκB-α degradation (a, a1). NF-κB p65 levels in the lung nuclear fractions were also significantly increased at 4 h after carrageenan injection compared to the sham-treated mice (b, b1). T0901317 treatment significantly reduced the levels of NF-κB p65 (b, b1). A representative blot of lysates obtained from 5 animals per group is shown and densitometry analysis of all animals is reported. The results in panel a1 and b1 are expressed as mean ± s.e.m. from n = 5/6 lung tissues for each group. ND: not detectable. *P < 0.01 versus sham group. °*P *< 0.01 versus carrageenan.

We evaluated IκB-α degradation and nuclear NF-κB p65 expression by Western blot analysis to investigate the cellular mechanisms whereby treatment with T0901317 attenuates the development of acute lung injury. Basal expression of IκB-α was detected in lung samples from sham-treated animals, whereas IκB-α levels were substantially reduced in lung tissues obtained from vehicle-treated animals at 4 h after carrageenan injection (Fig. 5a, see densitometry analysis 5a1). T0901317 (20 mg/kg) treatment prevented carrageenan-induced IκB-α degradation (Fig. 5a, see densitometry analysis 5a1). Moreover, NF-κB p65 levels in the lung nuclear fractions were also significantly increased at 4 h after carrageenan injection compared to the sham-treated mice (Fig. 5b, see densitometry analysis 5b1). T0901317 treatment significantly reduced the levels of NF-κB p65, as shown in Fig. 5b (see densitometry analysis 5b1).

### T0901317 modulates expression of Fas ligand after carrageenan injection

**Figure 6:**
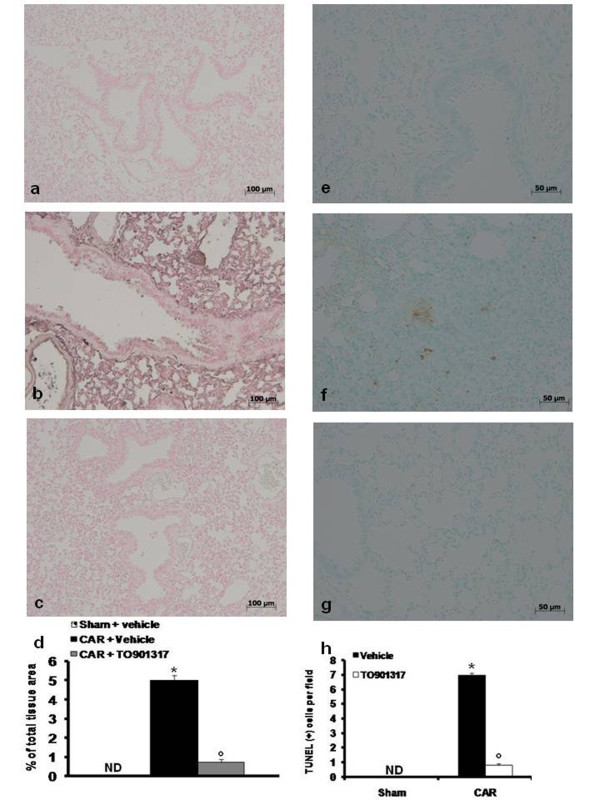
**Effect of T0901317 on carrageenan-induced Fas ligand expression and on apoptosis as measured by TUNEL like staining**. Positive staining for Fas ligand was observed in lung sections taken from carrageenan-treated mice pre-treated with vehicle (b, d) compared to sham-operated mice (a, d). In contrast, T0901317 (20 mg/kg) treatment reduced the degree of positive staining for FAS Ligand in the lung tissues (c, d). Positive TUNEL staining was observed in lung sections taken from carrageenan-treated mice pre-treated with vehicle (f, h). In contrast, tissue obtained from carrageenan treated mice pre-treated with T0901317 (20 mg/kg) demonstrated no apoptotic cells or fragments (g, h). Almost no apoptotic cells were observed in lungs of sham mice (e, h). The figure is representative of at least 3 experiments performed on different experimental days. ND: not detectable. Data are expressed as mean ± s.e.m. from n = 10 mice for each group. *P < 0.01 versus sham group. °*P *< 0.01 versus carrageenan.

Immunohistological staining for Fas ligand in the lung was also determined at 4 h after carrageenan injection. Lung sections from sham-treated mice did not stain for Fas ligand (Fig. 6a, see densitometry analysis 6d), whereas lung sections obtained from carrageenan-treated mice exhibited positive staining for Fas ligand (Fig. 6b, see densitometry analysis 6d) primarily localized in the inflammatory cells present in the lung tissue. T0901317 (20 mg/kg) treatment reduced the degree of positive staining for FAS Ligand in the lung tissues (Fig. 6c, see densitometry analysis 6d).

Effects of T0901317 on apoptosis in lung tissues after carrageenan-induced pleurisyTo investigate whether acute lung inflammation is associated with apoptotic cell death we measured TUNEL-like staining in lung tissues. At 4 hours after carrageenan administration, lung tissues demonstrated a marked appearance of dark brown apoptotic cells and intercellular apoptotic fragments (Fig. 6f, see 6h). In contrast, no apoptotic cells or fragments were observed in the tissues obtained from carrageenan-mice treated with T0901317 (Fig. 6g, see 6h). Similarly, no apoptotic cells were observed in lungs of sham-treated mice (Fig. 6e, see 6h).

### Western blot analysis and immunohistochemistry for Bax and Bcl-2

**Figure 7:**
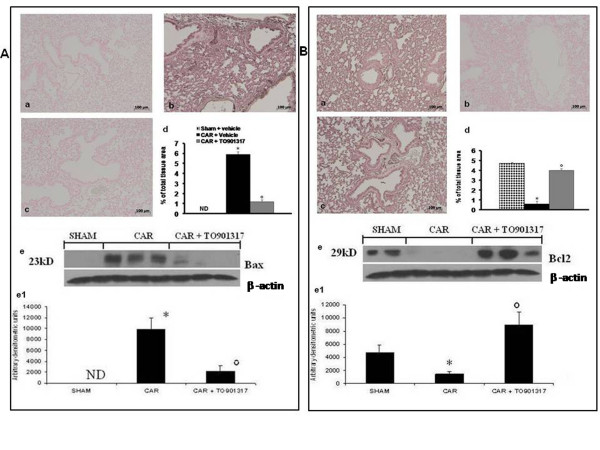
**Effect of T0901317 on carrageenan-induced Bax and Bcl-2 expression in the lung**. Representative Western blots showing no Bax expression in lung tissues obtained from sham-treated animals (Ae, Ae1). Bax levels were increased in the lung tissues from carrageenan-treated mice (Ae, Ae1). T0901317 (20 mg/kg) treatment prevented the carrageenan-induced Bax expression (Ae, Ae1). A basal level of Bcl-2 expression was detected in lung tissues from sham-treated mice (Be, Be1). At 4 hours after carrageenan administration, Bcl-2 expression was significantly reduced (Be, Be1). Treatment of mice with T0901317 (20 mg/kg) significantly attenuated carrageenan-induced inhibition of Bcl-2 expression (Be, Be1). A representative blot of lysates obtained from 5 animals per group is shown and densitometry analysis of all animals is reported. The results in panel Ae1 and Be1 are expressed as mean ± s.e.m. from n = 5/6 lung tissues for each group. *P < 0.01 versus sham group. °*P *< 0.01 versus carrageenan. Lung sections taken from carrageenan-treated mice pre-treated with vehicle showed positive staining for Bax (Ab, Ad) localized mainly in the inflammatory cells. The degree of positive staining for Bax was markedly reduced in lung sections obtained from mice pre-treated with 20 mg/kg T0901317 mice (Ac, Ad). Positive staining for Bcl-2 was observed in lung sections taken from sham mice (Ba, Bd). The degree of positive staining for Bcl-2 was markedly reduced in lung sections obtained from carrageenan-mice treated with vehicle (Bb, Bd). Pre-treatment with T0901317 20 mg/kg significantly attenuated the reduction in Bcl-2 expression caused by carrageenan (Bc, Bd). The figure is representative of at least 3 experiments performed on different experimental days. ND: not detectable Data are expressed as mean ± s.e.m. from n = 10 mice for each group. *P < 0.01 versus sham group. °*P *< 0.01 versus carrageena.

The presence of Bax in lung homogenates was investigated by Western blot 4 hours after carrageenan administration. No Bax expression was detected in lung tissues obtained from sham-treated animals (Fig. 7Ae, see densitometry analysis 7Ae1). Bax levels were substantially increased in the lung tissues from carrageenan-treated mice (Fig. 7Ae, see densitometry analysis 7Ae1). On the contrary, T0901317 (20 mg/kg) treatment prevented the carrageenan-induced Bax expression (Fig. 7Ae, see densitometry analysis 7Ae1).

To detect Bcl-2 expression, whole extracts from lung tissues of mice were also analyzed by Western blot analysis. A basal level of Bcl-2 expression was detected in lung tissues from sham-treated mice (Fig. 7Be, see densitometry analysis 7Be1). At 4 hours after carrageenan administration, Bcl-2 expression was significantly reduced (Fig. 7Be, see densitometry analysis 7Be1). Treatment of mice with T0901317 (20 mg/kg) significantly attenuated carrageenan-induced inhibition of Bcl-2 expression (Fig. 7Be, see densitometry analysis 7Be1).

Lung samples were also collected 4 hours after carrageenan administration in order to determine the immunohistological staining for Bax and Bcl-2. Lung tissues taken from sham-treated mice did not stain for Bax (Fig. 7Aa, see densitometry analysis 7Ad) whereas lung sections obtained from carrageenan-treated mice exhibited positive staining for Bax (Fig. 7Ab, see densitometry analysis 7Ad). T0901317 (20 mg/kg) treatment reduced the degree of positive staining for Bax in the lung of mice subjected to carrageenan-induced pleurisy (Fig. 7Ac, see densitometry analysis 7Ad).

In addition, lung sections from sham-treated mice demonstrated positive staining for Bcl-2 (Fig. 7Ba, see densitometry analysis 7Bd) whereas in carrageenan-treated mice Bcl-2 staining was significantly reduced (Fig. 7Bb, see densitometry analysis 7Bd). T0901317 (20 mg/kg) treatment significantly attenuated the loss of positive staining for Bcl-2 in mice subjected to carrageenan-induced pleurisy (Fig. 7Bc, see densitometry analysis 7Bd).

## Discussion

This study provides the evidence that T0901317 modulates: (i) the development of carrageenan-induced pleurisy, (ii) the infiltration of the lung with PMNs, (iii) the degree of lipid peroxidation in the lung, (iv) the expression of TNF-α and IL-1β, (v) iNOS expression (by immunohistochemistry and western blot analysis), (vi) the nitration of tyrosine residues, (vii) NF-κB expression (viii) Fas-ligand, (ix) apoptosis, (x) Bax and Bcl-2 expression and (xi) the degree of lung injury caused by injection of carrageenan. All of these findings support the view that T0901317 attenuates the degree of acute inflammation in the mouse. What, then, is the mechanism by which T0901317 reduces acute inflammation?

Liver × receptors (LXRs), a family of nuclear receptors, heterodimerize with retinoid × receptor (RXR) and bind specific elements (LXREs) characterized by direct repeats spaced by four nucleotides [29]. The LXR signaling pathway is thought to play an important role in lipid metabolism [30].

NF-κB plays a central role in the regulation of many genes responsible for the generation of mediators or proteins in inflammation. These include the genes for examples: TNF-α, IL-1β, iNOS and COX-2.

Recent data suggests that the LXR pathway antagonizes the NF-κB signaling pathway and inhibits the expression of inflammatory genes downstream of NF-κB [31]. Moreover, Joseph and colleagues have showed that a synthetic LXR agonist repressed the transcriptional activity of NF-κB, but not that of AP-1 in the presence of LXR, using a luciferase reporter assay [13]. Moreover, it has been, also, shown that the LXR agonist inhibits NF-κB-induced MMP-9 gene expression through LXR activation without affecting NF-κB DNA binding activity [32]We report here that carrageenan caused a significant increase in the IκB-α degradation in the lung tissues at 4 h, whereas treatment with the LXR agonist T0901317 significantly reduced this phosphorylation. Moreover, we also demonstrate that the selective and potent LXR agonist T0901317 inhibited the nuclear translocation of p65 in the lung tissues at 4 h after carrageenan administration. Taken together, the balance between pro-inflammatory and pro-survival roles of NF-κB may depend on the phosphorylation status of p65, and LXR receptor may play a central role in this process. However, the reasons for the apparent discrepancies in the modulatory effects of LXR receptor on NF-κB activity remain to be fully elucidated.

There is good evidence that TNF-α and IL-1β help to propagate the extension of a local or systemic inflammatory process. Various studies have clearly reported that inhibition of TNF-α formation significantly prevent the development of the inflammatory process [33]. This study demonstrates that T0901317 attenuates the TNF-α and IL-1β production in the pleural exudates of carrageenan-treated mice. Therefore, the inhibition of the production of TNF-α and IL-1β by T0901317 described in the present study is most likely attributed to the inhibitory effect the activation of NF-κB. Indeed, the effects of TO-901317 on TNFα parallel those previously reported in a rodent LPS lung injury model [10].

Furthermore, recently it has been demonstrated the expression of LXR in alveolar macrophages, alveolar type II cells, and PMNs and proceed to show potent anti-inflammatory and ant host defense effects of synthetic LXR agonists in the lung [10]. These anti-inflammatory and ant host defense effects share in common impairment of PMN recruitment to the lung [10].

In agreement with this previous observation, in the present study we have also demonstrated that T0901317 treatment significantly reduced the leukocyte infiltration as assessed by the specific granulocyte enzyme MPO at 4 hour after carrageenan administration. Activation and accumulation of leukocytes is one of the initial events of tissue injury due to release of oxygen free radicals, arachidonic acid metabolites and lysosomal proteases [20,34].

Enhanced formation of NO by iNOS may contribute to the inflammatory process [20,35]. This study demonstrates that T0901317 attenuates the expression of iNOS in the lung in carrageenan-treated mice. Therefore, the inhibition of iNOS expression by T0901317 described in the present study is most likely attributed to the inhibitory effect the activation of NF-κB. Moreover, the observed effect of T0901317 on iNOS expression is in agreement with a previous study in which Yasuda and colleagues have clearly described that another synthetic LXR agonist, 22R-HC inhibits NO production and iNOS expression in LPS-activated RAW264.7 macrophages suggesting that 22R-HC can negatively regulate excess NO during an inflammatory response, even after the onset of inflammation [31].

There is a large body of evidence showing that the production of reactive oxygen and nitrogen species play key roles in acute inflammation [36].

Nitrotyrosine formation, along with its detection by immunostaining, was initially proposed as a relatively specific marker for the detection of the endogenous formation "footprint" of peroxynitrite. There is, however, recent evidence that certain other reactions can also induce tyrosine nitration e.g. reaction of nitrite with hypoclorous acid and the reaction of MPO with hydrogen peroxide can lead to the formation of nitrotyrosine. Increased nitrotyrosine staining is therefore considered as an indicator of "increased nitrosative stress" rather than a specific marker of the generation of peroxynitrite.

We report here that carrageenan caused a significant increase in the nitrotyrosine formation and lipid peroxidation in the lung tissues at 4 h, which is significantly reduced by the treatment with the LXR agonist T0901317.

Therefore, the inhibition of nitrotyrosine formation and lipid peroxidation by T0901317 described in the present study is most likely attributed to the inhibitory effect the expression of iNOS by T09013178.

Generation of ROS have been implicated in induction of cell death and inflammation in the paw and lung tissues after carrageenan injection [20,37]. Furthermore, cell death induced by reactive oxygen species (ROS) depends on FasL expression mediated by redox sensitive activation of NF-κB [38]. FasL plays a central role in apoptosis induced by a variety of chemical and physical insults [39]. Recently it has been point out that Fas-Fas ligand (FasL) signaling plays a central role in acute inflammation (e.g. acute lung injury) [40,41]. We confirm here that the inflammatory process (carrageenan-induced pleurisy) leads to a substantial activation of FasL in the lung tissues which likely contribute in different capacities to the evolution of acute inflammation. In the present study, we found that FasL activation was significantly reduced in lungs from mice treated with T0901317. Moreover, in the present study we have also demonstrated that treatment with T0901317 attenuates the degree of apoptosis, measured by TUNEL detection kit, in the lung at 4 h after carrageenan administration. On the contrary some evidence have shown that chronic LXR activation is known to induce apoptosis in different cells line e.g. pancreatic β-cells [42].

Therefore, in the present study, we have identified pro-apoptotic transcriptional changes, including up-regulation of pro-apoptotic Bax and down-regulation of anti-apoptotic Bcl-2, using western blot assay and by immunohistochemical staining. We report in the present study for the first time that the treatment with the LXR agonist T0901317 in acute lung injury documents features of apoptotic cell death after carrageenan administration, suggesting that protection from apoptosis may be a prerequisite for anti-inflammatory approaches. In particular, we demonstrated that the treatment with T0901317 lowers the signal for Bax in treated group when compared with lung sections obtained from carrageenan-treated mice; while on the contrary, the signal is much more express for Bcl-2 in the LXR agonist T0901317 treated mice than in carrageenan-treated mice. This means that the LXR agonist T0901317 by inhibiting NF-κB prevents the loss of the anti-apoptotic way and reduced the pro-apoptotic pathway activation with a mechanism still to discover. Taken together, the results of the present study enhance our understanding of the role of the LXR receptor in the pathophysiology of acute inflammation. Our results imply that LXR agonists may be useful in the therapy of inflammation.

## Abbreviations

(L×R): Liver × receptor; (PMNs): neutrophils; (NOx): nitrite/nitrate; (TNF-α): tumor necrosis factor-α; (IL-1β): interleukin-1β; (NF-κB): transcription factor nuclear factor; (MPO activity): myeloperoxidase; (MDA levels): malondialdehyde; (iNOS): inducible nitric oxide synthase; (PBS): phosphate-buffer saline; (DMSO): dimethylsulfoxide; (NO): nitric oxide.

## Competing interests

The authors declare that they have no competing interests.

## Authors' contributions

CC carried out the experiment and drafted the manuscript. EM carried out the immunohistological studies. IP carried out the immunoassays. MG carried out the western blot analysis. PB participated in the design of the study. SC conceived the study, participated in its design and coordination and performed the statistical analysis. All authors read and approved the final manuscript.
